# Novel antimicrobial applications of copper oxide nanoparticles after combination with tissue conditioner used in complete prostheses

**DOI:** 10.1186/s12903-024-04534-w

**Published:** 2024-06-28

**Authors:** Saeed Nikanjam, Aria Yeganegi, Mohammad-Yousef Alikhani, Abbas Farmany, Seyed Amir Ghiasian, Roghayeh Hasanzade

**Affiliations:** 1 Department of Prosthodontics, School of Dentistry, Hamadan University of Medical Sciences, Hamadan, Iran; 2 Department of Microbiology, Faculty of Medicine, Hamadan University of Medical Sciences, Hamadan, Iran; 3 Dental Implant Research Center, School of Dentistry, Hamadan University of Medical Sciences, Hamadan, Iran; 4 Department of Medical Parasitology and Mycology Department, School of Medicine, Hamadan University of Medical Sciences and Health Services, Hamadan, Iran; 5 Department of Biostatistics, School of Public Health and Research Center for Health Sciences, Hamadan University of Medical Sciences, Hamadan, Iran

**Keywords:** Copper oxidenanoparticle, *C. Albicans*, Denture, *P. Aeruginosa*, *E. Faecalis*

## Abstract

**Background:**

Tissue conditioners are used for treating and improving the tissues supporting complete dentures. On the other hand, recent advances in nanotechnology have revolutionized various fields of science, including dentistry. The present study aimed to investigate **novel antimicrobial applications** of copper oxide nanoparticle-based tissue conditioner used in complete prostheses.

**Methods:**

The present experimental study included 126 tissue conditioner samples with different concentrations of copper oxide nanoparticles (20%, 10%, 5%, 2.5%, 1.25%, 0.625%, and 0% w/w). The samples were incubated with *Enterococcus faecalis*, *Pseudomonas aeruginosa*, and *Candida albicans* in 24-well plates for 24 h. Then, samples from the wells were re-incubated for 24 h, and the microorganisms were counted.

**Results:**

The culture media containing *E. faecalis* and *P. aeruginosa* showed significantly different growth between different nanoparticle concentrations following 24 h (*P* < 0.001), showing a reduction in bacterial growth with increased nanoparticle concentration. Both bacteria did not show any growth at the 20% concentration. However, *C. albicans* showed significant differences in growth between different nanoparticle concentrations following 48 h (*P* < 0.001), showing a reduction in growth with increased nanoparticle concentration. Also, the least growth was observed at the 20% concentration.

**Conclusions:**

In conclusion, the CuO nanoparticles were prepared using a green synthesis methon in the suitable sizes. Moreover, the tissue conditioners containing CuO nanoparticles showed acceptable antimicrobial properties against *E. faecalis*, *P. aeruginosa*, and *C. albicans*.

**Supplementary Information:**

The online version contains supplementary material available at 10.1186/s12903-024-04534-w.

## Background

Replacement of lost teeth is essential for health and high quality of life since edentulism can negatively affect facial aesthetics, speaking, and mastication [1]. There are different methods for replacing lost teeth, including implant-supported prostheses, implant-supported dental bridges, and removable prostheses [2, 3]. However, some of these options, such as dental implants, are less frequently used compared to other options due to limitations of the oral cavity and cost-ineffectiveness [4]. Considering the increased life expectancy of the middle-aged and the elderly, as well as the high prevalence of edentulism in this population, dental prostheses have become extensively popular in this age group. The prostheses used for tooth restoration should show enough biocompatibility in the oral cavity while improving facial aesthetics [5]. Moreover, prostheses should be properly designed in order to meet the physiological needs of the oral cavity, support the related soft and hard tissues without causing injuries, and have prolonged durability, thereby making the edentulous patients needless to new prostheses for several years [6].However, various bacterial and fungal species living in the oral cavity as the natural flora can turn into pathogens under certain conditions, such as prolonged use of dental prostheses. Thus, long-term use of these prostheses may result in stomatitis. Moreover, several factors, such as mucosal trauma, tobacco use, malignancies, endocrinopathic disorders, and the use of antibiotics, which can change the natural flora of the oral cavity, can predispose patients to prosthesis-induced stomatitis [7].On the other hand, tissue conditioners can be used for treating and improving the tissues supporting complete dentures. Lining the poor-fitting dentures helps in tissue healing and regeneration before molding for a new denture. Moreover, tissue conditioners can be used for temporary reasons, whether accessory or diagnostic, such as restoring the occlusal vertical dimensions and occlusal correction of old prostheses. Also, they can be used for evaluating the need for a permanent soft liner for patients with chronic or denture-induced pain [8].

Numerous efforts have been made to incorporate antimicrobial additives into the structures of tissue conditioners. These additives include antibiotics, essential oils, herbal oils, and notably, nanoparticles with antimicrobial properties [9]. Although some of these tissue conditioners show promising results against microorganisms, several deficiencies have been reported for the investigated cases. Among these defects, the lack of stability of the materials added to the tissue conditioner and the harmful effect on the mechanical properties of the tissue conditioner can be mentioned. Despite the positive effect of antimicrobial agents on tissue conditioners, there are no commercial antimicrobial tissue conditioners yet [9–13].

Nanotechnology has made significant advancements in various scientific domains, including dentistry, offering remarkable possibilities. One of the key attributes of nanoparticles is their high surface-to-volume ratio, which contributes to their exceptional properties [10]. Additionally, nanoparticles possess considerable strength and mechanical characteristics due to the formation of robust cross-links within polymer structures. Fragmenting materials into nanoparticles can be a potent method for creating structures with exceptionally high strength and excellent mechanical properties [11]. Furthermore, certain nanoparticles, such as silver, gold, copper, or zinc nanoparticles, exhibit antimicrobial properties [12, 13].

Despite their considerable optical, catalytic, electrical, and antifungal/antimicrobial properties, copper nanoparticles are less known in the field of nanotechnology compared to other nanoparticles [14]. However, multiple studies have shown their antimicrobial effects on human pathogens [15, 16]. Previous studies have introduced silver, zinc, or chitosan nanoparticles into the tissue conditioners’ structures to investigate their antimicrobial effects. However, despite their beneficial properties, copper nanoparticles are dramatically cost-effective, which justifies their use instead of other metal nanoparticles [17]. Considering the numerous shortcomings mentioned in relation to various substances added to tissue conditioners, the importance of the present study is to investigate the use of antimicrobial properties of copper nanoparticles in combination with tissue conditioners.

A study by Homsiang et al. used added zinc oxide nanoparticles to tissue conditioners, reporting their antifungal activity [18]. Moreover, Mousavi et al. have investigated the antimicrobial properties of silver, zinc, and chitosan nanoparticles [19, 20].

In dentistry, *Pseudomonas aeruginosa* infections often develop in patients with apical periodontitis and pulp necrosis [21, 22]. Moreover, *Enterococcus faecalis*, the predominant species of enterococcus genus in humans, is associated with several oral diseases, such as dental caries, root canal infections, periodontitis, and peri-implantitis [23, 24]. Also, immunocompromised individuals have increased colonization of *Candida albicans* in their oral cavity, leading to potential oral candidiasis [25–27].

To the best of our knowledge, no study has ever investigated the effect of adding copper oxide nanoparticles into the tissue conditioners’ structures on their antimicrobial properties. Thus, the present study aimed to investigate the antibacterial and antifungal properties of tissue conditioners used in complete prostheses following adding different ratios of copper oxide nanoparticles. The antimicrobial effects have been evaluated against *P. aeruginosa*, *E. faecalis*, and *C. albicans*.

## Materials and methods

### Sample size

In the present experimental study, the sample size was calculated at a minimum of 6 for each group using a confidence level of 95%, a statistical power of 80%, and the findings of previous studies [28, 29]. Thus, we used a total sample size of 126, considering 21 subgroups.

### Synthesis and characterization of copper oxide nanoparticle

The hydroalcoholic extract was prepared by grinding 20 g of propolis into a powder, which was then added to 100 mL of a hydroalcoholic solution (3:7 v/v) and kept at room temperature for one week. The hydroalcoholic solution, primarily composed of absolute ethanol, facilitated the extraction of polyphenolic compounds from the propolis, resulting in a higher extraction rate. After one week, the solution was filtered using a Whatman^®^ filter paper to remove any remaining propolis particles. The filtrate was then subjected to centrifugation at 4000 rpm to separate any solid particles. The resulting supernatant, free from solid particles, was preserved at 4 °C for future experiments. For the preparation of copper oxide nanoparticles, a solution containing 10 ppm of copper chloride was dissolved in deionized water. The copper chloride solution was mixed with the propolis extract solution at a temperature of 80 °C and stirred for 2 h at a uniform speed. The solution was filtered using a Whatman^®^ filter paper to eliminate impurities, followed by centrifugation at 4000 rpm. The precipitate obtained was isolated and purified. The supernatant from the previous centrifugation step was subjected to further centrifugation at 8000 rpm. The resulting precipitate was rinsed several times and utilized for the identification and characterization of the copper oxide nanoparticles [30, 31].

### Nanoparticle introduction into the structure of tissue conditioner

The present study used the TDV Soft Provisional tissue conditioner (TDV Dental Ltda, Brasil) to prepare samples with 20%, 10%, 5%, 2.5%, 1.25%, 0.625%, and 0% (w/w) copper oxide nanoparticle. The copper oxide nanoparticles were added to the tissue conditioner powder with a certain powder-to-solution ratio and were mixed for 30 s to become homogenized based on the manufacturer’s instructions. Then, the solution was poured into molds of equal sizes (diameter: 12 mm, depth: 2 mm). The mixed paste was placed between glass slides until it hardened [27]. Moreover, the samples with undesirable shapes, uneven surfaces, wrong powder-to-liquid ratios, and bubbles were excluded from the study.

### Microorganism culture

The present study evaluated the standard human pathogens, including *E. faecalis (*ATCC 29,212*)*, *P. aeruginosa* (ATCC 27,853), and *C. albicans (*ATCC 10,261*)*, which were obtained from the Microbial Bank of the Microbiology and Mycology Laboratory, School of Medicine, Hamedan University of Medical Sciences. The bacteria were cultured in blood agar media, while *C. albicans* was cultured in sabouraud dextrose agar media under laboratory conditions. A suspension with the concentration of 0.5 McFarland (1.5 × 10^8^ bacteria/mL) measured using a spectrophotometer (Spectrophotometer Single Beam AE-S60-4 V, A & E Lab co, UK) was prepared from the grown colonies and then was diluted to obtain a suspension in Mueller Hinton broth media with the concentration of 1.5 × 10^5^ bacteria/mL. Then, 200 µL of the suspension was added to tissue conditioner samples using a sampler (BRAND, Transferpette S, Germany). Afterward, the samples were incubated at a temperature of 37° C for 24 h, except for the media containing *C. albicans* that were incubated for 48 h.

### Microorganism growth assessment

The broth media containing the microbes on the tissue conditioners was sampled using a sterile swab, and the bacteria or fungi were cultured on blood agar or sabouraud dextrose agar media, respectively, using the lawn culture method. Following incubation for 24 h, the colonies were counted and reported using the CFU/mL.

### Nanoparticle characterization

The following methods were used for the characterization of nanoparticles:

1. X-Ray Diffraction (XRD): This method used the X-ray diffractometer (Xpert Pro MPD, Panalytical, Netherlands) at the wavelength of 1.5405 Å and the power of 40 KV/30 mA to evaluate the crystal structure of nanoparticles.
2. Fourier-Transform Infrared spectroscopy (FTIR): This method used the FTIR spectrometer (Spectrum400, PerkinElmer, USA) and also was conducted by KBR pellet technique under identical situations in the 500–4000 cm^− 1^ region.
3. Transmittance Electron Microscope (TEM): This device was used to examine the surface morphology and size of the nanoparticles. A transmittance electron microscope (TEM, Zeiss; EM10C model, Germany) at an accelerating voltage of 100 kv was used.

### Data analysis

Data analysis was performed using the SPSS software version 27 (SPSS Inc., Chicago, Illinois, United States). The mean and Standard Deviation (SD) of growth was calculated in each group. Then, the intergroup comparisons of the 24-hour growth of bacteria between different concentrations of copper oxide nanoparticles were performed using the one-way Analysis Of Variance (ANOVA). In case of significant differences, Turkey’s post hoc test was used to find certain concentrations with significantly different growth of the microorganism. Moreover, the intragroup comparison of *C. albicans* growth between 24-hour and 48-hour assessments was performed using the Mann-Whitney test. Also, the significance level was set at 0.05 for all tests, except for the post hoc tests, which had a significance level of 0.002.

## Results

### Copper oxide nanoparticle characterization

**Fig. 1:**
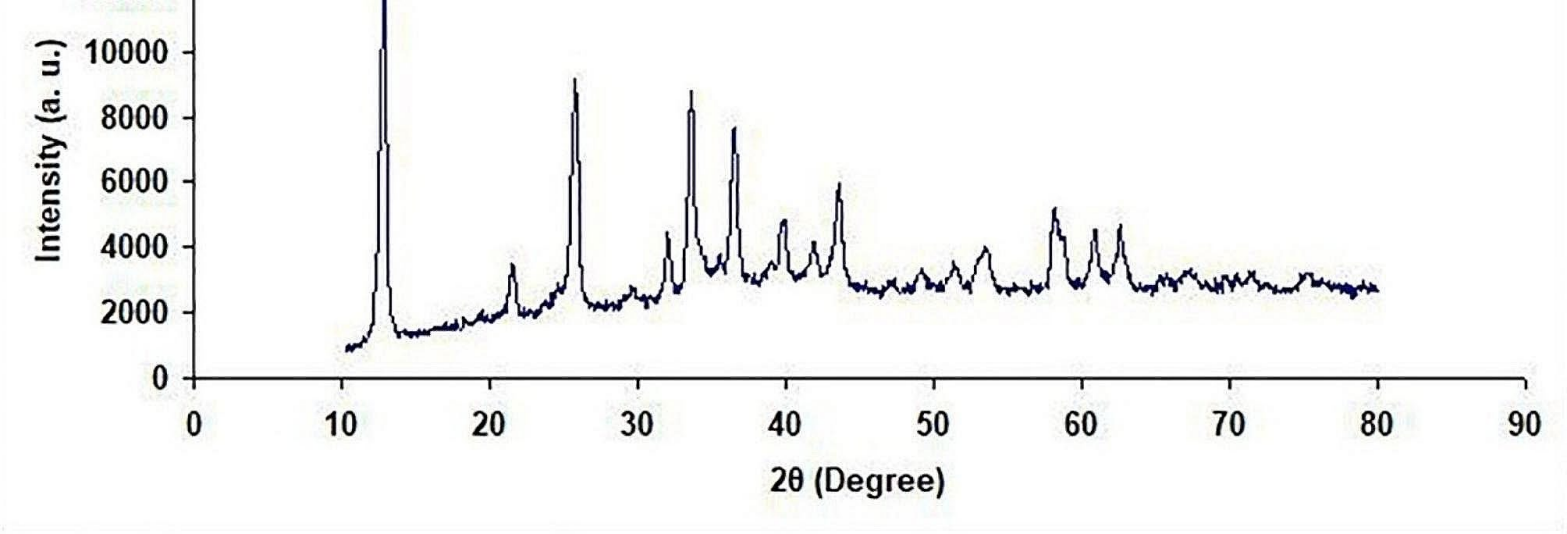
X-ray diffraction pattern of copper oxide nanoparticles

**Fig. 2:**
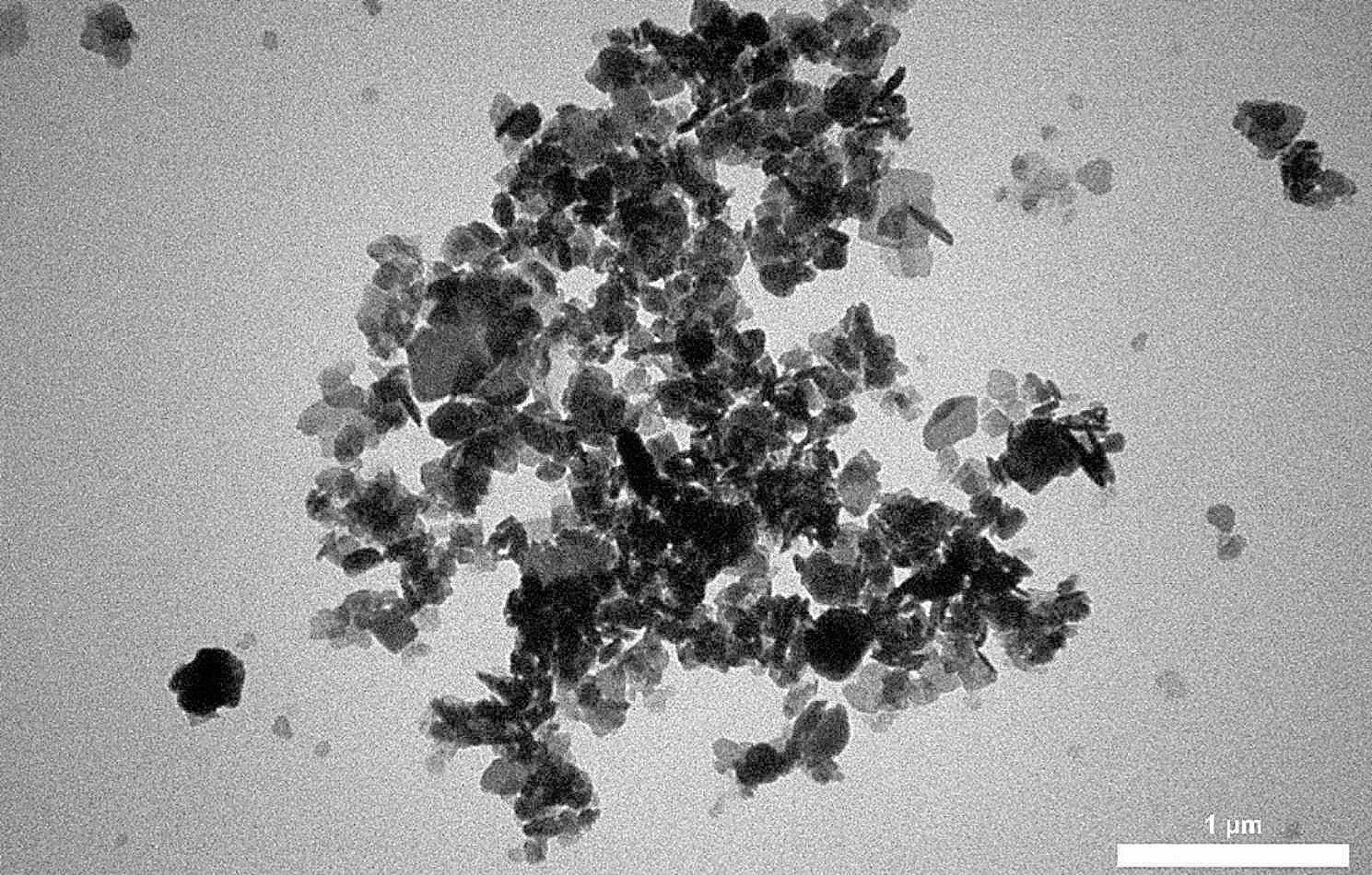
TEM image of copper oxide nanoparticles

Figure 1 presents the X-ray diffraction pattern of the copper oxide nanoparticles, showing monophasic nanoparticles with monoclinic structures. The peaks’ intensity and position in the obtained pattern completely correspond to the previously reported patterns. Figure 2 presents the TEM image taken from the copper oxide nanoparticles, showing crystalline copper oxide nanoparticles with a diameter of 30–70 nm.

**Fig. 3:**
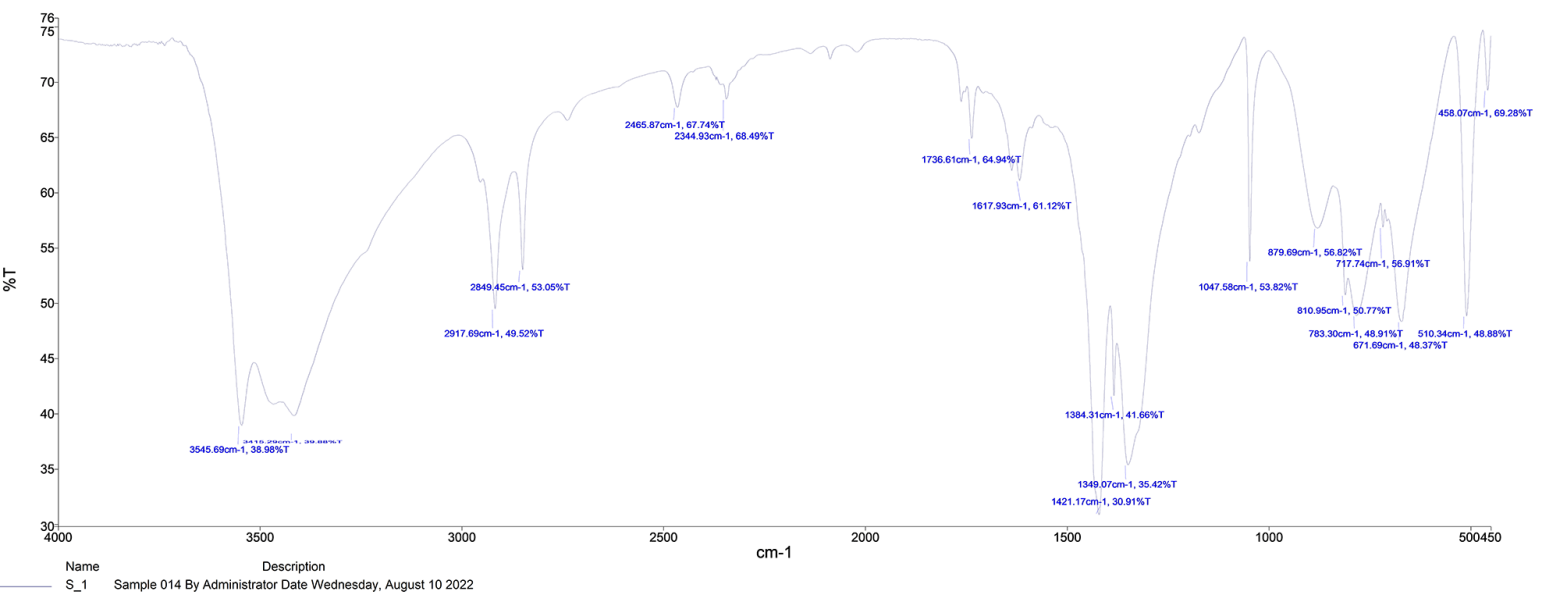
FTIR of copper oxide nanoparticles

According to the FTIR of copper oxide nanoparticles in Fig. 3, the half-broad band at about 3401 cm^− 1^ shows the stretching frequency of the hydroxyl group, an indicator of the surface morphology of the synthesized nanoparticles. Moreover, a peak in the 1047 cm^− 1^ corresponds to the bonds between copper and hydroxyl groups.

**Fig. 4:**
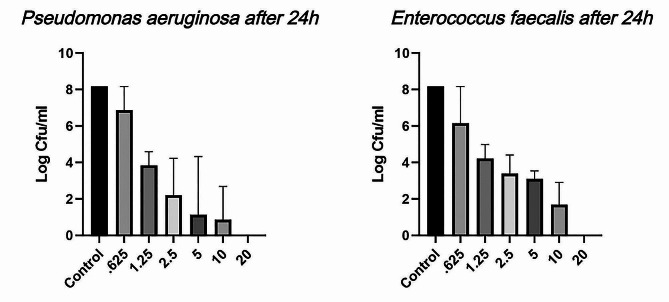
The mean bacterial growth in logarithm in different concentrations of copper oxide nanoparticles following 24 h

Table 1 presents the intergroup comparison of bacterial growth in different concentrations of copper oxide nanoparticles following 24 h, while Fig. 4 shows the mean bacterial growth in logarithm in different nanoparticle concentrations. According to Table 1, the culture media containing *E. faecalis* and *P. aeruginosa* showed significantly different growth between different nanoparticle concentrations (*P* < 0.001), showing a reduction in bacterial growth with increased nanoparticle concentration. Interestingly, both bacteria did not show any growth at the 20% concentration.

**Fig. 5:**
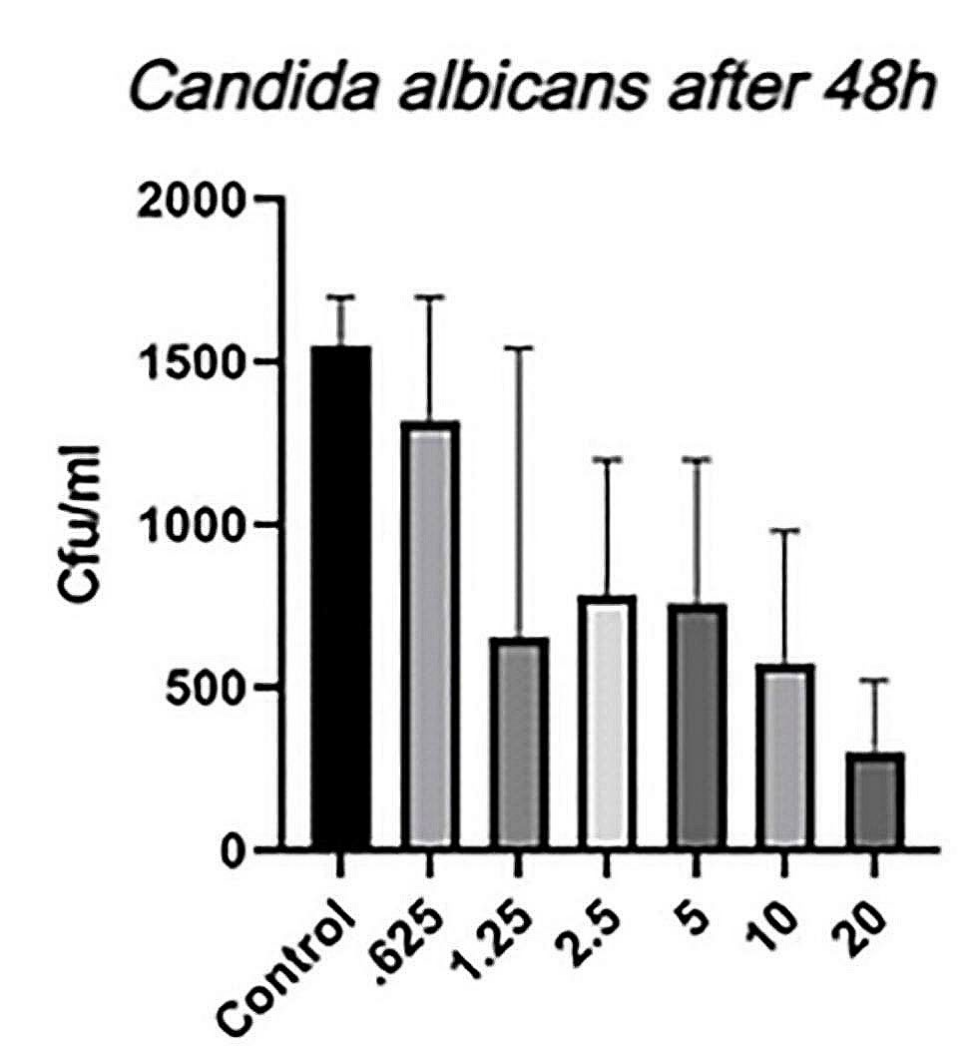
The mean growth of *C. albicans* in different concentrations of copper oxide nanoparticles following 48 h

Table 2 presents the intergroup and intragroup comparison of *C. albicans* growth in different concentrations of copper oxide nanoparticles following 24 and 48 h, while Fig. 5 shows the mean growth in different nanoparticle concentrations following 48 h. According to Table 2, C. *albicans* showed equal growth in all nanoparticle concentrations following 24 h, showing no significant difference (*P* > 0.05). However, the growth significantly reduced following 48 h of culture compared to the 24-hour assessment (*P* = 0.002), with the mean 24-hour growth being 9.9 × 10^4^ folds higher than the 48-hour growth. Moreover, the 48-hour growth was significantly different between different nanoparticle concentrations (*P* < 0.001), showing a reduction in growth with increased nanoparticle concentration. Thus, the least growth was observed at the 20% concentration.

Considering the significant intergroup differences in all studied pathogens calculated using the one-way ANOVA, pairwise comparisons were performed for each pathogen between different concentrations. Table 3 presents the pairwise intergroup comparisons using Tukey’s post hoc test. According to Table 3, culture media containing *E. faecalis* showed significant differences in 0-1.25%, 0-2.5%, 0-5%, 0-10%, 0-20%, 0.625-1.25%, 0.625-2.5%, 0.625-5%, 0.625-10%, and 0.625-20% pairwise comparisons following 24 h of culture (*P* < 0.001). Moreover, *P. aeruginosa* showed significantly different 24-hour growth in 0-0.625%, 0-1.25%, 0-2.5%, 0-5%, 0-10%, and 0-20% pairwise comparisons (*P* < 0.001). Also, *C. albicans* showed significantly different growth between the 0% and 20% concentrations (*P* < 0.001), as well as the 0.625% and 20% concentrations (*P* = 0.002). Thus, the growth was reduced in all pathogens with increased concentrations of copper oxide nanoparticles.

**Table 1:** Intergroup comparisons of bacterial growth in different concentrations of copper oxide nanoparticle following 24 h

CuO nanoparticle concentration	Bacterial Growth in 24 h (CFU/mL) ^a^	
	Enterococcus faecalis	Pseudomonas aeruginosa
0%	1.5 × 10^8^ ± 0	1.5 × 10^8^ ± 0
0.625%	1.0 × 10^8^ ± 7.7 × 10^7^	5.0 × 10^7^ ± 7.7 × 10^7^
1.25%	1.5 × 10^4^ ± 1.6 × 10^4^	3.6 × 10^4^ ± 3.4 × 10^4^
2.5%	3.4 × 10^3^ ± 6.7 × 10^3^	7.5 × 10^3^ ± 9.4 × 10^3^
5%	3.6 × 10^3^ ± 8.5 × 10^3^	2.1 × 10^3^ ± 1.5 × 10^3^
10%	1.3 × 10^2^ ± 2.2 × 10^2^	2.7 × 10^2^ ± 2.9 × 10^2^
20%	0	0
*P*-value ^b^	< 0.001*	< 0.001*

^a^ Presented as mean ± SD. ^b^ Calculated using the one-way ANOVA. * Significant difference (*P* < 0.05).

**Table 2:** Intergroup and intragroup comparisons of *C. Albicans* growth in different concentrations of copper oxide nanoparticle following 24 and 48 h

CuO nanoparticle concentration	Candida albicans Growth (CFU/mL) ^a^		Intragroup comparison	
	24-hour growth	48-hour growth	Mean difference	*P*-value ^c^
0%	1.0 × 10^5^ ± 0	1.6 × 10^3^ ± 1.6 × 10^2^	9.8 × 10^4^	0.002*
0.625%	1.0 × 10^5^ ± 0	1.3 × 10^3^ ± 5.1 × 10^2^	9.9 × 10^4^	0.002*
1.25%	1.0 × 10^5^ ± 0	6.6 × 10^2^ ± 6.2 × 10^2^	9.9 × 10^4^	0.002*
2.5%	1.0 × 10^5^ ± 0	7.8 × 10^2^ ± 4.2 × 10^2^	9.9 × 10^4^	0.002*
5%	1.0 × 10^5^ ± 0	7.6 × 10^2^ ± 4.0 × 10^2^	9.9 × 10^4^	0.002*
10%	1.0 × 10^5^ ± 0	5.8 × 10^2^ ± 3.1 × 10^2^	9.9 × 10^4^	0.002*
20%	1.0 × 10^5^ ± 0	3.0 × 10^2^ ± 1.6 × 10^2^	9.9 × 10^4^	0.002*
Intergroup comparison	1	< 0.001*		
*P*-value ^b^				

^a^ Presented as mean ± SD. ^b^ Calculated using the one-way ANOVA. ^c^ Calculated using the Mann-Whitney test. * Significant difference (*P* < 0.05).

**Table 3:** Pairwise intergroup comparison of microbial growth following 24–48 h

CuO nanoparticle concentration		Microbial Growth (CFU/mL)					
		Enterococcus faecalis (24 h)		Pseudomonas aeruginosa (24 h)		Candida albicans (48 h)	
		Mean difference	*P*-value ^a^	Mean difference	*P*-value ^a^	Mean difference	*P*-value ^a^
0%	0.625%	4.9 × 10^7^	0.074	9.9 × 10^7^	< 0.001*	2.3 × 10^2^	0.953
	1.25%	1.4 × 10^8^	< 0.001*	1.4 × 10^8^	< 0.001*	8.9 × 10^2^	0.008
	2.5%	1.4 × 10^8^	< 0.001*	1.4 × 10^8^	< 0.001*	7.7 × 10^2^	0.033
	5%	1.4 × 10^8^	< 0.001*	1.4 × 10^8^	< 0.001*	7.9 × 10^2^	0.025
	10%	1.4 × 10^8^	< 0.001*	1.4 × 10^8^	< 0.001*	9.7 × 10^2^	0.003
	20%	1.5 × 10^8^	< 0.001*	1.5 × 10^8^	< 0.001*	1.2 × 10^3^	< 0.001*
0.625%	1.25%	9.9 × 10^7^	< 0.001*	5.0 × 10^7^	0.072	6.6 × 10^2^	0.092
	2.5%	1.0 × 10^8^	< 0.001*	5.0 × 10^7^	0.071	5.4 × 10^2^	0.267
	5%	1.0 × 10^8^	< 0.001*	5.0 × 10^7^	0.071	5.6 × 10^2^	0.221
	10%	1.0 × 10^8^	< 0.001*	5.0 × 10^7^	0.071	7.4 × 10^2^	0.042
	20%	1.0 × 10^8^	< 0.001*	5.0 × 10^7^	0.071	1.0 × 10^3^	0.002*
1.25%	2.5%	1.1 × 10^4^	0.999	2.8 × 10^4^	0.999	1.3 × 10^2^	0.998
	5%	1.1 × 10^4^	0.999	3.3 × 10^4^	0.999	1 × 10^2^	0.999
	10%	1.5 × 10^4^	0.999	3.5 × 10^4^	0.999	8.0 × 10^1^	0.999
	20%	1.5 × 10^4^	0.999	3.6 × 10^4^	0.999	3.5 × 10^2^	0.730
2.5%	5%	1.2 × 10^2^	0.999	5.4 × 10^3^	0.999	2.5 × 10^1^	0.999
	10%	3.3 × 10^3^	0.999	7.2 × 10^3^	0.999	2.1 × 10^2^	0.972
	20%	3.4 × 10^3^	0.999	7.5 × 10^3^	0.999	4.8 × 10^2^	0.393
5%	10%	3.4 × 10^3^	0.999	1.8 × 10^3^	0.999	1.8 × 10^2^	0.985
	20%	3.6 × 10^3^	0.999	2.1 × 10^3^	0.999	4.6 × 10^2^	0.456
10%	20%	1.3 × 10^2^	0.999	2.6 × 10^2^	0.999	2.7 × 10^2^	0.898

^a^ Calculated using the Tukey’s post hoc test with Bonferroni’s adjustment. * Significant difference (*P* < 0.002).

## Discussion

The present study was the first to investigate the effect of tissue conditioners containing copper oxide nanoparticles on the growth of *E. faecalis* and *P. aeruginosa*.

The green biosynthesis of CuO nanoparticles was successfully conducted using a non-toxic, cost-effective, easy, and eco-friendly approach. These copper nanoparticles will probably be used in pharmaceutical formulations, drug delivery systems, and biomedical applications in the future since they can be prepared from natural products using a green biosynthesis method [32]. According to the findings from XRD, FTIR, and TEM investigations, the CuO nanoparticles made in the present study had monoclinic crystalline structures. Moreover, they had a suitable diameter in the nm range while maintaining their desirable properties and bonds.

Infection with *P. aeruginosa* is often reported in patients with apical periodontitis and pulp necrosis. Almost all patients with such infections are of lower socio-economic status and have poor oral and dental hygiene, gingivitis, and decayed teeth. The considerable resistance of *P. aeruginosa* to most antibiotics often makes its treatment extremely difficult, whether systematic or focal. In the present study, *P. aeruginosa* was used as a representative of gram-negative, antibiotic-resistant bacteria [22].

On the other hand, Enterococci are the causative agent of various infections, including endocarditis, meningitis, urinary tract, neonatal, and wound infections. Some of these infections are potentially fatal. Moreover, they are globally known as significant nosocomial pathogens, considering the growing emergence of antimicrobial-resistant phenotypes in the last few decades. Enterococci are resistant to vancomycin, tetracyclines, penicillins, cephalosporins, and aminoglycosides. Also, E. *faecalis* is often the cause of root canal treatment failure due to its high antibiotic resistance. In the present study, *E. faecalis* is the representative of gram-positive, antibiotic-resistant bacteria [23, 24].

Considering the movement limitations of elderly patients using removable prostheses, [6–8] one of the clinical applications of tissue conditioners containing CuO nanoparticles is to help these patients prevent the growth of pathogenic microorganisms, which is facilitated the observance of hygiene by patients.

Previous research has reported the reactive oxygen species production and resultant oxidative stress as the potential cause of the antibacterial properties of copper nanoparticles. Moreover, these nanoparticles can show direct cytotoxicity by disrupting the membrane function, altering its permeability, and attacking different cellular structures and proteins containing phosphorus and sulfur [25]. It is worth mentioning that the present study did not use CuO concentrations higher than 20% since they exert cytotoxic effects. Moreover, we used the principles of the Minimal Inhibitory Concentration (MIC) technique to dilute the CuO nanoparticle concentration by half in each step [19, 33]. Also, according to Chul Lee et al. the no-observed-adverse-effect levels of Cu nanoparticles and Cu micro particles were determined to be 100 and ≥ 400 mg/kg/day, respectively [33]. As nanoparticles are solid and in combination with tissue conditioner in our study, they are much harmless compared to the previous study that nanoparticles were used in solution form.

The present study used the colony counting method to assess the number of microorganisms, which is superior to the optical absorption density method since it does not count the non-viable microorganisms [34]. The reduced growth of *E. faecalis* and *P. aeruginosa* in different concentrations of CuO nanoparticles confirmed the benefit of this method. Moreover, the increasing concentration of CuO nanoparticles could directly reduce bacterial growth. Also, the 20% CuO nanoparticle concentration completely stopped the growth in both studied bacteria.

A study by Maqusood et al. evaluated the antimicrobial effect of CuO nanoparticles, reporting compatible results with the present study regarding the effect of these nanoparticles on *E. faecalis* and *P. aeruginosa*. Moreover, its effect on *E. faecalis* was comparable with streptomycin as the standard positive control [35]. Also, another study by Mardones et al. used CuO nanoparticles inside the root canal to suppress bacterial growth. Furthermore, they conducted an in vitro assessment of *E. faecalis* growth, reporting a dramatically reduced growth following 24 h compared to the negative control group, which was compatible with our findings. It seems that CuO nanoparticles have higher inhibitory effects on bacterial growth compared to Cu particles with conventional dimensions due to increased bacterial exposure to Cu and facilitated penetration into the cells of the microorganisms [36].

On the other hand, a study by Mousavi et al. used ZnO-Ag-based tissue conditioners to inhibit the growth of *E. faecalis* and *P. aeruginosa*, reporting complete growth arrest in 20% nanoparticle concentration. This study was compatible with the present study regarding the nanoparticle concentration and incubation duration. Thus, it can be concluded that ZnO-Ag and CuO nanoparticles have equal antimicrobial effects [27]. Moreover, another study investigated the antimicrobial effect of chitosan-based tissue conditioners, reporting complete growth arrest in 5% and 10% chitosan nanoparticle concentrations for *P. aeruginosa* and *E. faecalis*, respectively. Thus, it can be concluded that chitosan nanoparticles have a higher inhibitory effect on the growth of these two bacteria compared to CuO nanoparticles [20]. Also, a study by García Marin et al. compared the antimicrobial effect of Cu nanoparticles on *C. albicans* compared to common drugs used for treating *C. albicans* infections, including fluconazole, nystatin, and amphotericin B, reporting a higher antifungal effect for CuO nanoparticles compared to amphotericin B. Furthermore, the observed effect was highly concentration-dependent [37]. Thus, the mentioned study was compatible with our findings.

Geographically, propolis samples exhibit distinct chemical compositions that directly influence their antioxidant properties. For instance, ethanolic extracts of propolis from Russia and Italy demonstrate similar antioxidant effects due to the presence of shared polyphenols. In contrast, Brazilian propolis has a relatively lower antioxidant effect owing to its diminished polyphenol content [38]. However, the current understanding of the properties of Iranian propolis is limited and incomplete. Further research is required to comprehensively explore its therapeutic potential. The utilization of propolis is driven by its well-documented therapeutic properties, aiming to augment the economic value of raw propolis and facilitate the development of innovative pharmaceuticals [39].

According to Amiri et al. study, [40] copper nanoparticles have a preventive effect on infections caused by different species of Candida. Also, according to the study of Garcia-Marin et al. [37], copper oxide nanoparticles have a very high effect as a topical antifungal treatment against Candida albicans.

According to the findings of this study on the antimicrobial effects of CuO nanoparticles, it is recommended to do more research regarding the addition of these nanoparticles in heat-cured and 3D-printed denture base resins.

In the end, it is recommended to conduct further studies on the physical and mechanical properties of CuO nanoparticle-based tissue conditioners. Moreover, the antimicrobial effects of such tissue conditioners should be investigated on a more extensive range of microorganisms found in the oral cavity. More research regarding antibiotic resistance tests and biofilm formation is also recommended.

## Conclusion

In the present study, the CuO nanoparticles were made properly in suitable sizes. Moreover, the tissue conditioners containing copper oxide nanoparticles showed acceptable antimicrobial effects against *E. faecalis*, *P. aeruginosa*, and *C. albicans*. Also, it is recommended to conduct further studies on this topic to find the optimal concentration of CuO nanoparticles in tissue conditioners, thereby introducing the application of nanoparticles to the field of dental material science to make commercial tissue conditioners containing copper oxide nanoparticles.

### Electronic supplementary material

Below is the link to the electronic supplementary material.

Supplementary Material 1

## Data Availability

The datasets used and/or analysed during the current study available from the corresponding author on reasonable request.All data generated or analysed during this study are included in this published article [and its supplementary information files].

## References

[1] Wöstmann B, Budtz-Jørgensen E, Jepson N, Mushimoto E, Palmqvist S, Sofou A et al. Indications for removable partial dentures: a literature review. Int J Prosthodont. 2005;18(2). 15889662

[2] Tyson K, Yemm R, Scott B. Understanding partial denture design. Oxford University Press; 2006.

[3] Grossmann Y, Nissan J, Levin L (2009). Clinical effectiveness of implant-supported removable partial dentures—a review of the literature and retrospective case evaluation. J Oral Maxillofac Surg.

[4] Sharma A, Shashidhara H (2014). A review: flexible removable partial dentures. J Dent Med Sci.

[5] Geramiuanah F, ASADI G. Post insertion problems of removable prosthesis: Causes, diagnosis and treatment. 2007.

[6] Carr AB, Brown DT. McCracken’s Removable Partial Prosthodontics-E-Book. Elsevier Health Sciences; 2010.

[7] Khasawneh S, al-Wahadni A (2002). Control of denture plaque and mucosal inflammation in denture wearers. J Ir Dent Assoc.

[8] Zarb GA (2013). Prosthodontic treatment for edentulous patients: complete dentures and implant-supported prostheses.

[9] Iqbal Z, Zafar MS (2016). Role of antifungal medicaments added to tissue conditioners: a systematic review. J Prosthodontic Res.

[10] Zhu A, Cai A, Zhou W, Shi Z (2008). Effect of flexibility of grafted polymer on the morphology and property of nanosilica/PVC composites. Appl Surf Sci.

[11] Sun L, Gibson RF, Gordaninejad F, Suhr J (2009). Energy absorption capability of nanocomposites: a review. Compos Sci Technol.

[12] Fernando S, Gunasekara T, Holton J. Antimicrobial Nanoparticles: applications and mechanisms of action. 2018.

[13] Wang L, Hu C, Shao L (2017). The antimicrobial activity of nanoparticles: present situation and prospects for the future. Int J Nanomed.

[14] Ramyadevi J, Jeyasubramanian K, Marikani A, Rajakumar G, Rahuman AA (2012). Synthesis and antimicrobial activity of copper nanoparticles. Mater Lett.

[15] Meto A, Colombari B, Sala A, Pericolini E, Meto A, Peppoloni S (2019). Antimicrobial and antibiofilm efficacy of a copper/calcium hydroxide-based endodontic paste against Staphylococcus aureus, Pseudomonas aeruginosa and Candida albicans. Dent Mater J.

[16] Xu VW, Nizami MZI, Yin IX, Yu OY, Lung CYK, Chu CH (2022). Application of copper nanoparticles in dentistry. Nanomaterials.

[17] Bogdanović U, Lazić V, Vodnik V, Budimir M, Marković Z, Dimitrijević S (2014). Copper nanoparticles with high antimicrobial activity. Mater Lett.

[18] Homsiang W, Kamonkhantikul K, Arksornnukit M, Takahashi H (2021). Effect of zinc oxide nanoparticles incorporated into tissue conditioner on antifungal, physical, and mechanical properties. Dent Mater J.

[19] Mousavi SA, Khorramdel A, Aghajanzadeh H (2020). Evaluation of antifungal and antibacterial properties of adding Ag, ZnO, Chitosan nanoparticles to tissue conditioners of complete dentures. Res Dent Sci.

[20] Mousavi SA, Ghotaslou R, Kordi S, Khoramdel A, Aeenfar A, Kahjough ST (2018). Antibacterial and antifungal effects of chitosan nanoparticles on tissue conditioners of complete dentures. Int J Biol Macromol.

[21] Nair VV, Karibasappa GN, Dodamani A, Prashanth VK (2016). Microbial contamination of removable dental prosthesis at different interval of usage: an in vitro study. J Indian Prosthodont Soc.

[22] Nord CE, Sjöberg L, Wadström T (1972). Pseudomonas Aeruginosa in oral infections. Acta Odontol Scand.

[23] Komiyama EY, Lepesqueur LS, Yassuda CG, Samaranayake LP, Parahitiyawa NB, Balducci I (2016). Enterococcus species in the oral cavity: prevalence, virulence factors and Antimicrobial susceptibility. PLoS ONE.

[24] Patel M (2022). Oral cavity and Candida albicans: Colonisation to the development of infection. Pathogens.

[25] Applerot G, Lellouche J, Lipovsky A, Nitzan Y, Lubart R, Gedanken A (2012). Understanding the Antibacterial mechanism of CuO nanoparticles: revealing the Route of Induced oxidative stress. Small.

[26] Nam KY, Lee CJ, Subramani K, Ahmed W (2019). Chapter 14 - characterization of silver nanoparticles incorporated acrylic-based tissue conditioner with antimicrobial effect and cytocompatibility. Nanobiomaterials in Clinical Dentistry.

[27] Mousavi SA, Ghotaslou R, Akbarzadeh A, Azima N, Aeinfar A, Khorramdel A (2019). Evaluation of antibacterial and antifungal properties of a tissue conditioner used in complete dentures after incorporation of ZnO–Ag nanoparticles. J Dent Res Dent Clin Dent Prospects.

[28] Nam K-Y (2011). In vitro antimicrobial effect of the tissue conditioner containing silver nanoparticles. J Adv Prosthodont.

[29] Haghdoost A, Baneshi M, Marzban M (2011). How to Estimate the Sample size in Special conditions? (part two). Iran J Epidemiol.

[30] Habibipour R, Moradi-Haghgou L, Farmany A (2019). Green synthesis of AgNPs@PPE and its Pseudomonas aeruginosa biofilm formation activity compared to pomegranate peel extract. Int J Nanomed.

[31] https://www.thejcdp.com/doi/JCDP/pdf/10.5005/jp-journals-10024-3393.

[32] Hajizadeh YS, Harzandi N, Babapour E, Yazdanian M, Ranjbar R (2022). Green synthesize and characterization of copper nanoparticles using Iranian Propolis extracts. Adv Mater Sci Eng.

[33] Lee I-C, Ko J-W, Park S-H, Shin N-R, Shin I-S, Moon C (2016). Comparative toxicity and biodistribution assessments in rats following subchronic oral exposure to copper nanoparticles and microparticles. Part Fibre Toxicol.

[34] Pan H, Zhang Y, He GX, Katagori N, Chen H (2014). A comparison of conventional methods for the quantification of bacterial cells after exposure to metal oxide nanoparticles. BMC Microbiol.

[35] Ahamed M, Alhadlaq HA, Khan MAM, Karuppiah P, Al-Dhabi NA (2014). Synthesis, characterization, and antimicrobial activity of copper oxide nanoparticles. J Nanomaterials.

[36] Ml JM, C G, Covarrubias DCG. C. In Vitro Antibacterial Properties of Copper Nanoparticles as Endodontic Medicament against Enterococcus faecalis. 2018.

[37] Garcia-Marin LE, Juarez-Moreno K, Vilchis-Nestor AR, Castro-Longoria E (2022). Highly antifungal activity of Biosynthesized Copper Oxide nanoparticles against Candida albicans. Nanomaterials.

[38] Freires IA, de Alencar SM, Rosalen PL (2016). A pharmacological perspective on the use of Brazilian Red Propolis and its isolated compounds against human diseases. Eur J Med Chem.

[39] Zabaiou N, Fouache A, Trousson A, Baron S, Zellagui A, Lahouel M, Lobaccaro J-M (2017). Biological properties of propolis extracts: something new from an ancient product. Chem Phys Lipids.

[40] Amiri M, Etemadifar Z, Daneshkazemi A, Nateghi M (2017). Antimicrobial effect of copper oxide nanoparticles on some oral bacteria and candida species. J Dent Biomaterials.

