# Supplementary material for: Novel antimicrobial applications of copper oxide nanoparticles after combination with tissue conditioner used in complete prostheses
Source: BMC Oral Health. 2024 Jun 28;24:752. doi: 10.1186/s12903-024-04534-w (PMC11214236; doi:10.1186/s12903-024-04534-w)
Supplement: Supplementary file 1 — Supplementary Material 1 [file 12903_2024_4534_MOESM1_ESM.docx]

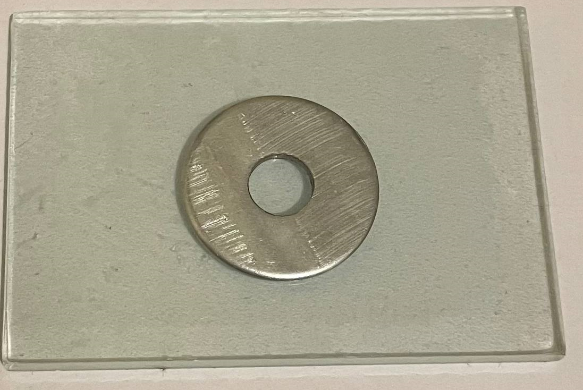


**Figure a. Metal mold samples**


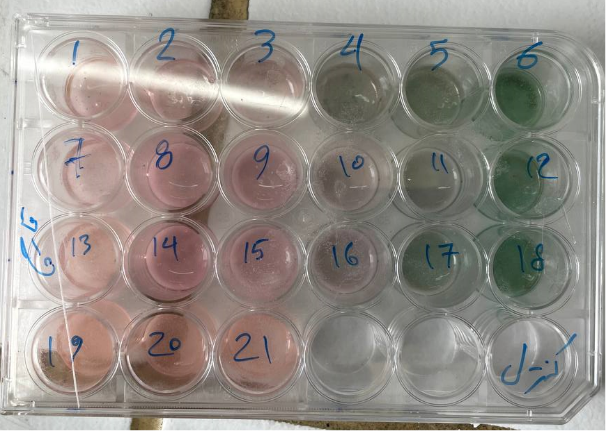


**Figure b. Micro-plate containing tissue conditioner samples**


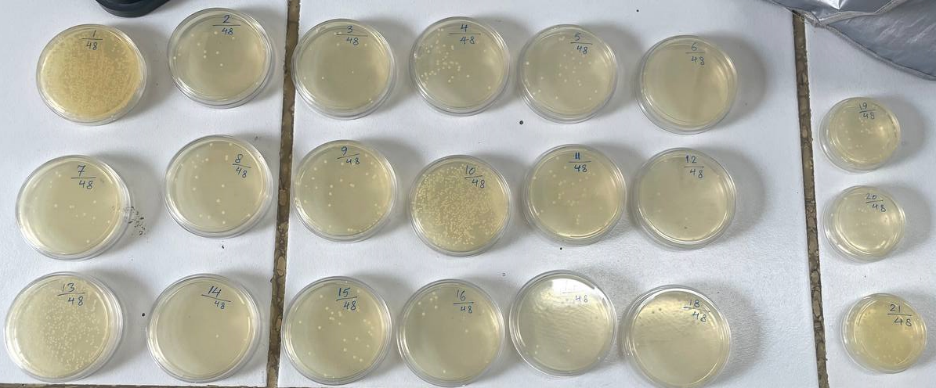


**Figure c. Cultivated plates**

S1. Intergroup comparisons of bacterial growth in different concentrations of copper oxide nanoparticle following 24 hours.

| CuO nanoparticle concentration | Bacterial Growth in 24 hours (CFU/mL) ^a^ | |
| --- | --- | --- |
|  | *Enterococcus faecalis* | *Pseudomonas aeruginosa* |
| 0% | 1.5×10^8^ ± 0 | 1.5×10^8^ ± 0 |
| 0.625% | 1.0×10^8^ ± 7.7×10^7^ | 5.0×10^7^ ± 7.7×10^7^ |
| 1.25% | 1.5×10^4^ ± 1.6×10^4^ | 3.6×10^4^ ± 3.4×10^4^ |
| 2.5% | 3.4×10^3^ ± 6.7×10^3^ | 7.5×10^3^ ± 9.4×10^3^ |
| 5% | 3.6×10^3^ ± 8.5×10^3^ | 2.1×10^3^ ± 1.5×10^3^ |
| 10% | 1.3×10^2^ ± 2.2×10^2^ | 2.7×10^2^ ± 2.9×10^2^ |
| 20% | 0 | 0 |
| P-value ^b^ | <0.001* | <0.001* |

^a^ Presented as mean±SD. ^b^ Calculated using the one-way ANOVA. * Significant difference (P<0.05).

S2. Intergroup and intragroup comparisons of *C. albicans* growth in different concentrations of copper oxide nanoparticle following 24 and 48 hours.

| CuO nanoparticle concentration | *Candida albicans* Growth (CFU/mL) ^a^ | | Intragroup comparison | |
| --- | --- | --- | --- | --- |
|  | 24-hour growth | 48-hour growth | Mean difference | P-value ^c^ |
| 0% | 1.0×10^5^ ± 0 | 1.6×10^3^ ± 1.6×10^2^ | 9.8×10^4^ | 0.002* |
| 0.625% | 1.0×10^5^ ± 0 | 1.3×10^3^ ± 5.1×10^2^ | 9.9×10^4^ | 0.002* |
| 1.25% | 1.0×10^5^ ± 0 | 6.6×10^2^ ± 6.2×10^2^ | 9.9×10^4^ | 0.002* |
| 2.5% | 1.0×10^5^ ± 0 | 7.8×10^2^ ± 4.2×10^2^ | 9.9×10^4^ | 0.002* |
| 5% | 1.0×10^5^ ± 0 | 7.6×10^2^ ± 4.0×10^2^ | 9.9×10^4^ | 0.002* |
| 10% | 1.0×10^5^ ± 0 | 5.8×10^2^ ± 3.1×10^2^ | 9.9×10^4^ | 0.002* |
| 20% | 1.0×10^5^ ± 0 | 3.0×10^2^ ± 1.6×10^2^ | 9.9×10^4^ | 0.002* |
| Intergroup comparison | 1 | <0.001* |  |  |
| P-value ^b^ |  |  |  |  |

^a^ Presented as mean±SD. ^b^ Calculated using the one-way ANOVA. ^c^ Calculated using the Mann-Whitney test. * Significant difference (P<0.05).

S3. Pairwise intergroup comparison of microbial growth following 24 or 48 hours.

| CuO nanoparticle concentration | | Microbial Growth (CFU/mL) | | | | | |
| --- | --- | --- | --- | --- | --- | --- | --- |
|  |  | *Enterococcus faecalis* (24 h) | | *Pseudomonas aeruginosa* (24 h) | | *Candida albicans* (48 h) | |
|  |  | Mean difference | P-value ^a^ | Mean difference | P-value ^a^ | Mean difference | P-value ^a^ |
| 0% | 0.625% | 4.9×10^7^ | 0.074 | 9.9×10^7^ | <0.001* | 2.3×10^2^ | 0.953 |
|  | 1.25% | 1.4×10^8^ | <0.001* | 1.4×10^8^ | <0.001* | 8.9×10^2^ | 0.008 |
|  | 2.5% | 1.4×10^8^ | <0.001* | 1.4×10^8^ | <0.001* | 7.7×10^2^ | 0.033 |
|  | 5% | 1.4×10^8^ | <0.001* | 1.4×10^8^ | <0.001* | 7.9×10^2^ | 0.025 |
|  | 10% | 1.4×10^8^ | <0.001* | 1.4×10^8^ | <0.001* | 9.7×10^2^ | 0.003 |
|  | 20% | 1.5×10^8^ | <0.001* | 1.5×10^8^ | <0.001* | 1.2×10^3^ | <0.001* |
| 0.625% | 1.25% | 9.9×10^7^ | <0.001* | 5.0×10^7^ | 0.072 | 6.6×10^2^ | 0.092 |
|  | 2.5% | 1.0×10^8^ | <0.001* | 5.0×10^7^ | 0.071 | 5.4×10^2^ | 0.267 |
|  | 5% | 1.0×10^8^ | <0.001* | 5.0×10^7^ | 0.071 | 5.6×10^2^ | 0.221 |
|  | 10% | 1.0×10^8^ | <0.001* | 5.0×10^7^ | 0.071 | 7.4×10^2^ | 0.042 |
|  | 20% | 1.0×10^8^ | <0.001* | 5.0×10^7^ | 0.071 | 1.0×10^3^ | 0.002* |
| 1.25% | 2.5% | 1.1×10^4^ | 0.999 | 2.8×10^4^ | 0.999 | 1.3×10^2^ | 0.998 |
|  | 5% | 1.1×10^4^ | 0.999 | 3.3×10^4^ | 0.999 | 1×10^2^ | 0.999 |
|  | 10% | 1.5×10^4^ | 0.999 | 3.5×10^4^ | 0.999 | 8.0×10^1^ | 0.999 |
|  | 20% | 1.5×10^4^ | 0.999 | 3.6×10^4^ | 0.999 | 3.5×10^2^ | 0.730 |
| 2.5% | 5% | 1.2×10^2^ | 0.999 | 5.4×10^3^ | 0.999 | 2.5×10^1^ | 0.999 |
|  | 10% | 3.3×10^3^ | 0.999 | 7.2×10^3^ | 0.999 | 2.1×10^2^ | 0.972 |
|  | 20% | 3.4×10^3^ | 0.999 | 7.5×10^3^ | 0.999 | 4.8×10^2^ | 0.393 |
| 5% | 10% | 3.4×10^3^ | 0.999 | 1.8×10^3^ | 0.999 | 1.8×10^2^ | 0.985 |
|  | 20% | 3.6×10^3^ | 0.999 | 2.1×10^3^ | 0.999 | 4.6×10^2^ | 0.456 |
| 10% | 20% | 1.3×10^2^ | 0.999 | 2.6×10^2^ | 0.999 | 2.7×10^2^ | 0.898 |

^a^ Calculated using the Tukey's post hoc test with Bonferroni's adjustment. * Significant difference (P<0.002).
